# Ginsenoside Rg3 Improved Age-Related Macular Degeneration Through Inhibiting ROS-Mediated Mitochondrion-Dependent Apoptosis In Vivo and In Vitro

**DOI:** 10.3390/ijms252111414

**Published:** 2024-10-24

**Authors:** Rui-Yi Hu, Si-Min Qi, Ya-Jun Wang, Wen-Lin Li, Wan-Chen Zou, Zi Wang, Shen Ren, Wei Li

**Affiliations:** 1 College of Chinese Medicinal Materials, Jilin Provincial International Joint Research Center for the Development and Utilization of Authentic Medicinal Materials, Jilin Agricultural University, Changchun 130118, China; hry221510@163.com (R.-Y.H.); ms77_1998@163.com (S.-M.Q.); wangyj559@jlau.edu.cn (Y.-J.W.); liwenlin9974@163.com (W.-L.L.); wanchenzou@163.com (W.-C.Z.); z.wang@jlau.edu.cn (Z.W.); 2 College of Life Sciences, Jilin Agricultural University, Changchun 130118, China

**Keywords:** ginsenoside Rg3, age-related macular degeneration, sodium iodate, retinal pigment epithelium, apoptosis

## Abstract

Age-related macular degeneration (AMD) is marked by a progressive loss of central vision and is the third leading cause of irreversible blindness worldwide. The exact mechanisms driving the progression of this macular degenerative condition remain elusive, and as of now, there are no available preventative measures for dry AMD. According to ancient records, ginseng affects the eyes by brightening them and enhancing wisdom. Modern pharmacological research shows that the active ingredients in ginseng, ginsenosides, may be used to prevent or improve eye diseases that threaten vision. Some articles have reported that ginsenoside Rg3 can treat diabetic retinopathy in mice, but no reports exist on its effects and mechanisms in AMD. Therefore, the role and mechanism of ginsenoside Rg3 in AMD warrant further study. This study aims to investigate the effects of Rg3 on AMD and its underlying molecular mechanisms. We established a mouse model of AMD to examine the impact of ginsenoside Rg3 on NaIO_3_-induced apoptosis in the retina and to explore the related intrinsic mechanisms. The in vivo results indicated that ginsenoside Rg3 prevents NaIO_3_-induced apoptosis in retinal pigment epithelial cells by inhibiting reactive oxygen species production and preventing the reduction in mitochondrial membrane potential. Additionally, we assessed the levels of protein expression within the apoptosis pathway. Ginsenoside Rg3 decreased the expression of Bax, cleaved caspase-3, and cleaved caspase-9 proteins. Additionally, it increased the expression of Bcl-2 by decreasing P-JNK levels. Moreover, our in vivo results showed that ginsenoside Rg3 enhanced retinal structure, increased the relative thickness of the retina, and decreased the extent of disorganization in both the inner and outer nuclear layers. Ginsenoside Rg3 may safeguard the retina against NaIO_3_-induced cell apoptosis by attenuating reactive-oxygen-species-mediated mitochondrial dysfunction, in which the JNK signaling pathway is also involved. These findings suggest that ginsenoside Rg3 has the potential to prevent or attenuate the progression of AMD and other retinal pathologies associated with NaIO_3_-mediated apoptosis.

## 1. Introduction

Age-related macular degeneration (AMD) is a form of progressive degenerative retinopathy, and it is the primary cause of impaired vision and blindness in the elderly population worldwide. Anti-vascular endothelial growth factor (anti-VEGF) therapy is the primary method employed to manage wet AMD. Nevertheless, there are presently no viable treatment alternatives accessible for dry AMD. This is mainly due to the intricate pathogenesis of dry AMD, which encompasses numerous cellular pathways [1]. The main approach for treating wet AMD is the use of anti-vascular endothelial growth factor (anti-VEGF) therapy. However, there are currently no effective treatment options available for dry AMD. This is primarily due to the complex pathogenesis of dry AMD, which involves multiple cellular pathways [2]. Dry AMD is distinguished by the existence of drusen and the accelerated degeneration of the retinal pigment epithelium (RPE), particularly in the macular region, which is clinically known as geographic atrophy, leading to the gradual deterioration of vision. Multiple risk factors have been linked to AMD, encompassing older age, oxidative stress, genetic predisposition, persistent inflammation, smoking, and ethnicity [3]. The onset of AMD is primarily linked to the exposure of the retina to an excessive amount of reactive oxygen species (ROS) [4]. Excessive ROS production disrupts the normal operations of RPE cells, hindering their antioxidant defenses, metabolic functions, and lysosomal degradation capabilities, ultimately leading to cell apoptosis [5].

Oxidative stress plays a central role in the initiation and progression of retinal disorders. It elevates intracellular ROS levels, leading to retinal damage, and is a primary pathological factor [6]. ROS accelerate cellular aging and diminish mitochondrial function in RPE cells, which results in cellular damage [7]. A recent study demonstrated that oxidative stress has a significant role in the development, degeneration, and dysfunction of RPE, as well as its age-related loss [8]. Repeated exposure to oxidative stress from ROS, such as sodium iodate (NaIO_3_), causes RPE damage [9]. NaIO_3_, an oxidative toxic agent, causes selective RPE cell damage and can reportedly serve as a reproducible in vitro and in vivo model of AMD [10]. 

**Figure 1:**
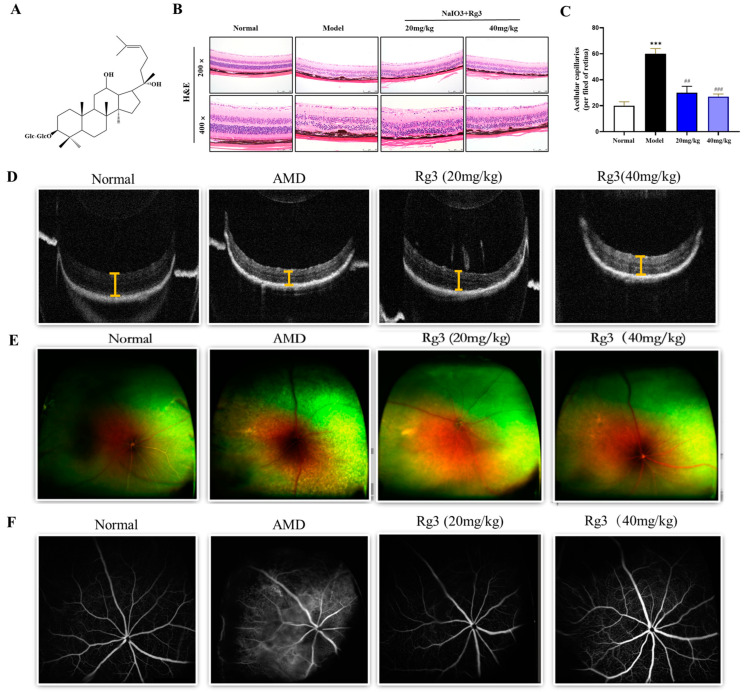
The structure of ginsenoside Rg3 (**A**). Representative H&E-stained retinal sections from the four groups were obtained between 600 and 900 μm from the optic nerve in both the superior and inferior hemiretina (**B**). Quantitative analysis of the number of acellular capillaries per field in retinas (**C**). The protective effects of ginsenoside Rg3 on retinal thickness in NaIO_3_-treated mice were evaluated using OCT, the length of the yellow section marks the thickness of the mouse retina (**D**). Retinal photography was used to assess the protective effects of ginsenoside Rg3 on NaIO_3_-treated mice (**E**). Fundus fluorescein angiography (FFA) was employed to determine the protective role of ginsenoside Rg3 in NaIO_3_-treated mice (**F**). Note: Values are expressed as mean ± S.D. *** *p* < 0.001 vs. normal group; ^##^ *p* < 0.01, ^###^ *p* < 0.001 vs. AMD group (*n* = 3).

Ginseng has been known since ancient times for its efficacy in improving vision. It has been shown to possess neuroprotective properties, which could help in preserving retinal ganglion cells and maintaining visual function in glaucoma patients. Ginsenoside Rg3 (S-Rg3), an essential bioactive compound derived from the roots of *Panax ginseng*, plays significant roles in anti-cancer and anti-inflammatory effects (Figure 1A) [11,12]. In relation to cataracts, Rg3 has exhibited antioxidant properties that may have the potential to reduce oxidative-stress-induced harm to the eye’s lens [13,14]. This antioxidative effect might play a role in slowing down the advancement of cataract development [15]. Moreover, in the context of diabetic retinopathy, ginsenoside Rg3 has been shown to reduce the expression of TNF-α and VEGF in ganglion cells [16]. Rg3 has demonstrated potential in mitigating oxidative stress and inflammation, both of which play significant roles in the onset and advancement of diabetic retinopathy [17]. Its capacity to facilitate the regeneration and restoration of retinal cells may additionally contribute to alleviating diabetic retinopathy [18]. However, it is important to note that despite the positive effects observed in glaucoma, cataracts, and diabetic retinopathy, the applications of Rg3 against AMD have not been fully revealed.

## 2. Results

### 2.1. Protective Effects of Rg3 on Retinal Degeneration

To verify the correlation between the in vitro and in vivo studies on NaIO_3_-induced retinal damage, we established a mouse model of retinal degeneration. After a single administration of NaIO_3_, optical coherence tomography (OCT) was used to assess the thickness of the retina, including the inner nuclear layer (INL) and outer nuclear layer (ONL). In the present study, OCT images from the mice treated with NaIO_3_ on day 7 revealed a significant decrease in total retinal thickness compared to the mice in the mock group. There were some small particles beneath the RPE, clinically similar to drusen. However, the treatment with Rg3 demonstrated a significant ability to restore retinal thickness and reduce the accumulation of drusen deposits beneath the RPE. Representative images of retinal sections from the four study groups are presented (Figure 1D). The thicknesses of the whole retina, INL, and ONL were quantified. In the mice treated with NaIO_3_, the thicknesses of the total retina, INL, and ONL were notably decreased compared to the control group. In addition, the NaIO_3_-treated mice exhibited disruptions at the inner- and outer-segment junctions (IS/OS) of the photoreceptors.

To assess the retinal damage further visually in the AMD mouse model, we used H&E staining, as shown in Figure 1B,C, and evaluated the therapeutic effect of ginsenoside Rg3. In the control group, the retinal layers exhibited organized and uniform cell arrangements. In contrast, both the model group and the treatment group showed wavy and irregular cell alignment in the INL and IOL, displaying signs of disarray. Additionally, the RPE layer in some areas exhibited disruptions and losses. In summary, the retinal structures of the model and treatment groups showed varying degrees of abnormalities compared to the control group, and noticeable disruptions and depositions of brown–black particles were observed in the RPE layer. The ginsenoside Rg3 treatment group demonstrated reduced severity of RPE layer disruptions and ONL folding compared to the sodium iodate model group, with fewer RPE elevations. Moreover, the cell density of both the INL and the ONL in the treatment group was greater than that in the sodium iodate model group.

To visually assess the therapeutic effect of ginsenoside Rg3 on AMD, we conducted fundus photography and FFA experiments on AMD mice. As shown in Figure 1D, the fundus photography revealed a significant number of yellow–white deposits in the retina of the AMD model group mice, while the mice treated with ginsenoside Rg3 showed a noticeable reduction in yellow–white precipitates. This observation was further confirmed by FFA examination. The AMD mice exhibited disruption of the inner and outer retinal barriers, fluorescent leakage, and an unclear optic disc, indicating retinal RPE structural disorganization and atrophy. However, this phenomenon was reversed after the administration of ginsenoside Rg3 (Figure 1E,F).

### 2.2. Rg3 Inhibited the Apoptosis Signaling Pathway in the AMD Model

**Figure 2:**
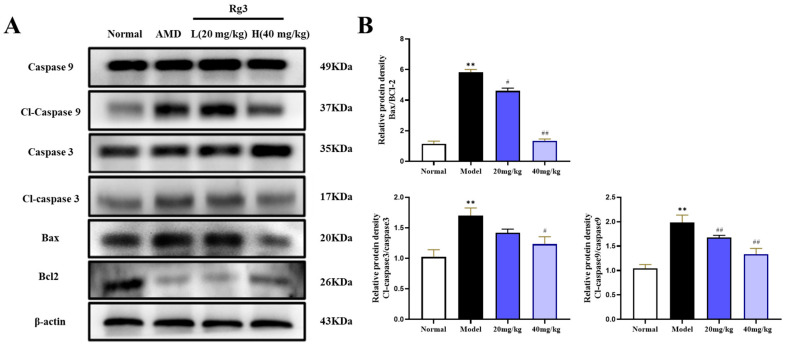
The influence of ginsenoside Rg3 administration on the modulation of apoptosis-related protein levels (**A**). Examination of protein expression associated with apoptosis (**B**). Note: Values are expressed as mean ± S.D. ** *p* < 0.01 vs. normal group; ^#^ *p* < 0.05, ^##^ *p* < 0.01 vs. AMD group (*n* = 3).

To delve into the specifics of apoptosis, we employed a Western blot analysis to examine the expression patterns of the pro-apoptotic and anti-apoptotic factors across the different experimental groups. As depicted in Figure 2, a significant upregulation of the expression of pro-apoptotic proteins Bax, as well as the activated forms of cleaved caspase-3 and cleaved caspase-9, was observed in the AMD model group treated with NaIO_3_ when compared to the normal control group. Concurrently, the expression of the anti-apoptotic protein Bcl-2 significantly decreased (*p* < 0.01). However, following treatment with ginsenoside Rg3 at doses of 20 mg/kg and 40 mg/kg, there was a dose-dependent reversal in the expression levels of these apoptosis-related proteins. These findings suggest that ginsenoside Rg3 exhibits a dose-dependent protective effect against NaIO_3_-induced retinal damage.

### 2.3. Rg3 Diminished JNK Signaling and P-JNK Expression in an AMD Model

**Figure 3:**
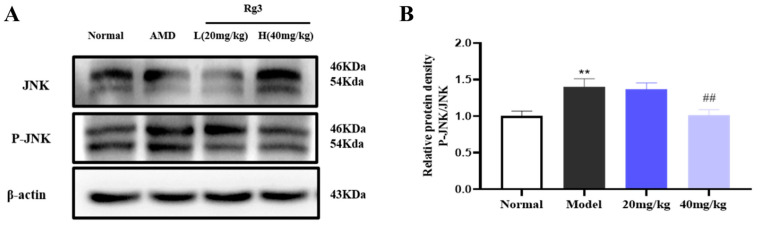
JNK protein levels after in vivo treatment with diverse concentrations of ginsenoside Rg3 and NaIO_3_ (**A**). Statistical scanning of JNK expression (**B**). All values are expressed as mean ± S.D., ** *p* < 0.01 vs. normal group, ^##^ *p* < 0.01 vs. AMD group (*n* = 3).

Previous studies have indicated that ginsenoside Rg3 exerts an inhibitory effect on the ROS-mediated JNK signaling pathway. To investigate whether the JNK signaling pathway is involved in the protective effects of ginsenoside Rg3 against NaIO_3_-induced retinal damage, a Western blot analysis was employed to determine the expression of JNK-signaling-pathway-related proteins in vivo following NaIO_3_ induction. As shown in Figure 3, the level of JNK phosphorylation in the NaIO_3_ group significantly increased compared to the normal group (*p* < 0.01). Interestingly, the pretreatment with ginsenoside Rg3 reduced the expression of p-JNK in the mice challenged with NaIO_3_ (*p* < 0.01). These findings suggest that ginsenoside Rg3 may mitigate NaIO_3_-induced retinal damage in vivo by inhibiting the JNK signaling pathway. In summary, ginsenoside Rg3 may protect against NaIO_3_-induced apoptosis in vivo by inhibiting JNK activation.

### 2.4. Effect of Rg3 on NaIO_3_-Induced Apoptosis in RPE Cells

**Figure 4:**
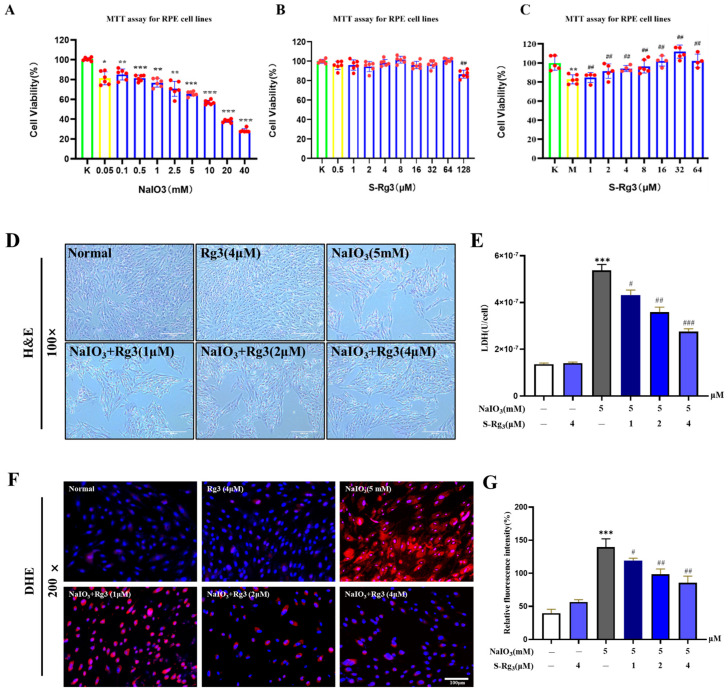
The effects of NaIO_3_ on RPE cell activity (**A**). Effect of ginsenoside Rg3 on the activity of RPE cells (**B**). Effect of ginsenoside Rg3 on NaIO_3_-induced RPE cytotoxicity (**C**); H&E staining of RPE cells induced by NaIO_3_ (**D**). The level of LDH released in RPE cells induced by NaIO_3_ (**E**). Effects of ginsenoside Rg3 with different concentrations (1, 2, and 4 μM) on ROS generation in NaIO_3_-induced RPE cells (**F**). The relative fluorescence density of ROS, the blue fluorescence is DAPI, and the red fluorescence is ROS. (**G**). All values are expressed as mean ± S.D. * *p* < 0.05, ** *p* < 0.01, *** *p* < 0.001 vs. normal group; ^#^ *p* < 0.05, ^##^ *p* < 0.01 ^###^ *p* < 0.001 vs. NaIO_3_ group (*n* = 3).

NaIO_3_ was used to investigate the mechanism of NaIO_3_ damage to RPE cells and the potential protective mechanism of ginsenoside Rg3. The MTT viability assay was used to evaluate the effects of NaIO_3_ or ginsenoside Rg3 on RPE cells. As depicted in Figure 4A, the decrease in RPE cell viability after 24 h of exposure to NaIO_3_ was concentration-dependent (*p* < 0.01). The cytocidal rate of NaIO_3_ at a concentration of 5 mM was 70% when compared to the control group (*p* < 0.001). Figure 4B illustrates that ginsenoside Rg3, within the concentration range of 0.5 to 128 μM, exhibited no evident cytotoxicity. Furthermore, Figure 4C shows that pretreatment with ginsenoside Rg3 at concentrations between 0.5 and 64 μM successfully countered the reduction in cell viability caused by NaIO_3_ in a manner that depended on the concentration used (*p* < 0.01). Compared to the NaIO_3_ group, ginsenoside Rg3 at concentrations of 1, 2, and 4 μM showed a protective effect on cells within a specific concentration range (*p* < 0.01 or *p* < 0.001). These results indicate that ginsenoside Rg3 offers protection to cells against damage caused by NaIO_3_ in a manner that depends on its concentration.

Cellular pathological changes and their variations were examined using H&E staining (Figure 4D). In the control group, the cells exhibited a uniform and regular spindle-shaped morphology with strong proliferative capacity, and the cell nuclei were mostly located in the center of the cells. In the NaIO_3_ group, a significant amount of cell death was observed, with irregular and clustered cell distribution, increased cell transparency, and nuclear membrane and nucleolus fragmentation. After treatment with different doses of ginsenoside Rg3, the cellular state gradually approached that of the control group, indicating that ginsenoside Rg3 effectively inhibited the damage caused by NaIO_3_.

Furthermore, to mitigate the potential impact of varying cell numbers on our experimental outcomes, we opted to employ the release of LDH as a metric for assessing the extent of oxidative damage to the cell membrane (normalized to total cell count). As illustrated in Figure 4E, in comparison to the control group, the NaIO_3_-treated RPE cells exhibited a marked and statistically significant increase in LDH release (*p* < 0.01), underscoring the substantial damage inflicted upon RPE cells by NaIO_3_. However, following exposure to different concentrations of ginsenoside Rg3, we observed varying degrees of reduction in LDH release by RPE cells, signifying the protective effect of ginsenoside Rg3 on RPE cell membrane integrity. The primary reason for NaIO_3_ toxicity is the excessive buildup of ROS. The concentration of ROS was assessed using DHE, the production of ROS in RPE cells at different concentrations was determined, and the fluorescence intensity was measured. Following the combined treatment of ginsenoside Rg3 with varying concentrations and NaIO_3_, it was observed that ginsenoside Rg3 significantly reduced ROS accumulation in cells (*p* < 0.01) (Figure 4F,G).

### 2.5. Rg3 Inhibited NaIO_3_-Induced Apoptosis in RPE Cells

**Figure 5:**
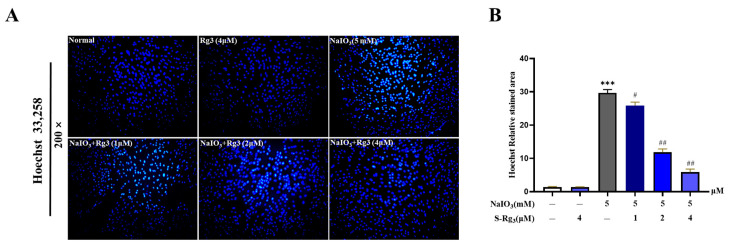

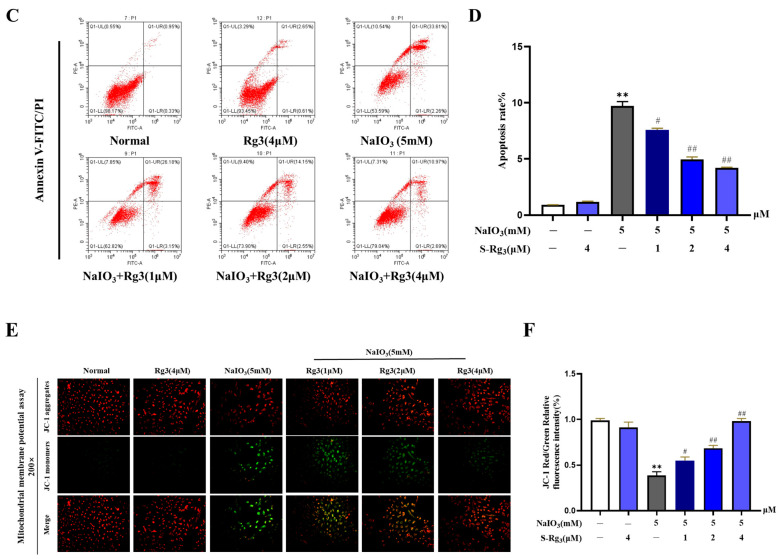
Inhibitory effect of ginsenoside Rg3 on NaIO_3_-induced apoptosis of RPE cells (**A**). The fluorescence levels were measured (**B**). Flow cytometry was utilized to examine the impact of ginsenoside Rg3 on NaIO_3_−induced apoptosis in RPE cells (**C**) and the percentage of apoptosis (**D**). The effect of ginsenoside Rg3 on the inhibition of mitochondrial membrane potential induced by NaIO_3_ in RPE cells was assessed (**E**), and the fluorescence intensities were measured and quantified (**F**). All values are expressed as mean ± S.D. ** *p* < 0.01, *** *p* < 0.001 vs. normal group; ^#^ *p* < 0.05, ^##^ *p* < 0.01 vs. NaIO_3_ group (*n* = 3).

To investigate the ability of ginsenoside Rg3 to mitigate NaIO_3_-induced apoptosis, we used Hoechst 33,258 fluorescence staining to observe changes in RPE cell apoptosis (Figure 5A,B). Compared to the control group, the cells exposed to NaIO_3_ displayed characteristic signs of apoptosis, including nuclear condensation, nuclear membrane fragmentation, intense blue staining of chromatin, and a substantial presence of apoptotic bodies. However, the 24 h preincubation with ginsenoside Rg3 improved these apoptosis characteristics. These findings strongly suggest that ginsenoside Rg3 can alleviate NaIO_3_-induced apoptosis in RPE cells. To further corroborate the protective effect of ginsenoside Rg3 on NaIO_3_-induced apoptosis in RPE cells, we assessed the apoptosis rate using flow cytometry (Figure 5C,D). The flow cytometry analysis indicated that apoptosis was low at both the initial and later stages. By contrast, after NaIO_3_ stimulation, the overall apoptosis rate increased significantly (*p* < 0.01). However, the pretreatment with ginsenoside Rg3 led to a decrease in the percentage of RPE cells undergoing early and late apoptosis when compared to the group treated with NaIO_3_ (*p* < 0.05 or *p* < 0.01). These findings offer additional support for the possible role of ginsenoside rg3 in protecting against NaIO_3_-induced apoptosis in RPE cells.

A decrease in mitochondrial membrane potential is a key indicator of the early phases of apoptosis. JC-1 staining was conducted to examine how ginsenoside Rg3 influences the alterations in mitochondrial membrane potential of RPE cells caused by NaIO_3_ (Figure 5E,F). The fluorescence staining revealed that the NaIO_3_ treatment significantly reduced the mitochondrial membrane potential in the RPE cells compared to the control group. However, preincubation with ginsenoside Rg3 at concentrations of 1, 2, and 4 μM for 24 h reversed this decline in mitochondrial membrane potential, suggesting that ginsenoside Rg3 can protect mitochondrial function by inhibiting the NaIO_3_-induced reduction in mitochondrial membrane potential in RPE cells.

### 2.6. Rg3 Inhibited the Apoptosis Signaling Pathway in RPE Cells

**Figure 6:**
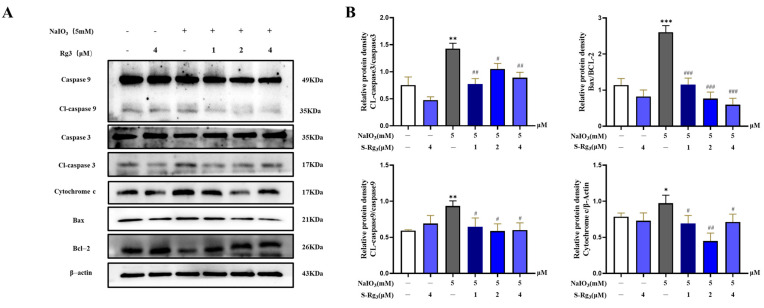
The effect of ginsenoside Rg3 treatment on the improvement of apoptosis protein levels (**A**). Apoptosis protein expression analysis (**B**). Note: Values are expressed as mean ± S.D. * *p* < 0.05, ** *p* < 0.01, *** *p* < 0.001 vs. normal group; ^#^ *p* < 0.05, ^##^ *p* < 0.01, ^###^ *p* < 0.001 vs. NaIO_3_ group (*n* = 3).

In order to provide a more comprehensive understanding of the extent of apoptosis, we conducted a Western blot analysis to evaluate the expression levels of pro-apoptotic and anti-apoptotic factors across all the experimental groups. As demonstrated in Figure 6, the expression levels of pro-apoptotic proteins, including Bax, cleaved caspase-3, cleaved caspase-9, and cytochrome C, were significantly elevated in the RPE cells following stimulation with NaIO_3_. Conversely, the expression of the anti-apoptotic protein Bcl-2 significantly decreased in the NaIO_3_-treated RPE cells compared to the control group (*p* < 0.05). The expression levels of the aforementioned apoptosis-related proteins exhibited a dose-dependent reversal following a 24 h pretreatment with ginsenoside Rg3 at concentrations of 1, 2, and 4 μM, in comparison to cells treated with NaIO_3_. These findings suggest that ginsenoside Rg3 exerts a protective effect against NaIO_3_-induced apoptosis in RPE cells in a dose-dependent manner.

### 2.7. Rg3 Reduced JNK Signaling Pathways and P-JNK Protein Expression in RPE Cells

**Figure 7:**
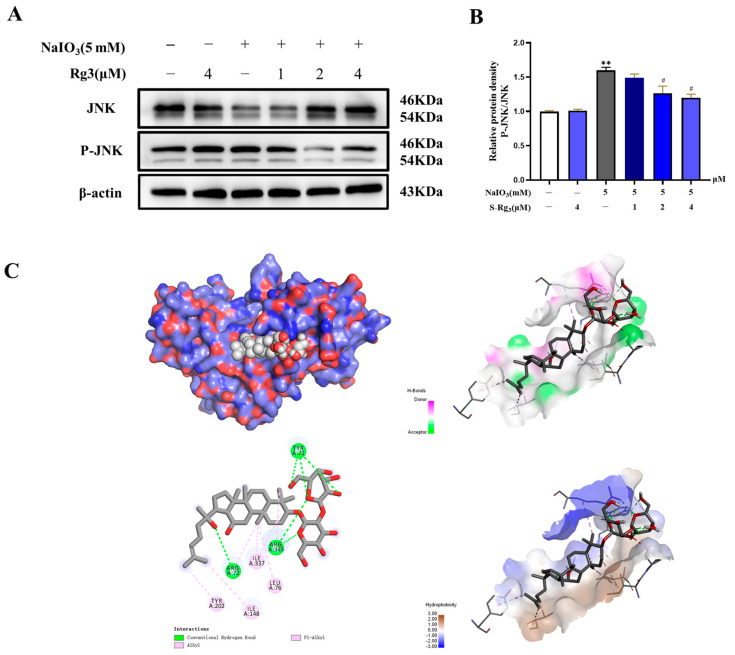
Expression levels of JNK proteins after in vitro treatment with different concentrations of ginsenoside Rg3 and NaIO_3_ (**A**). Scanning quantitative analysis of JNK (**B**). Molecular docking pattern of Rg3-JNK (**C**). All values were expressed as mean ± S.D., ** *p* < 0.01 vs. normal group, ^#^ *p* < 0.05 vs. NaIO_3_ group (*n* = 3).

We examined the expression of JNK pathway proteins in RPE cells using Western blot analysis. As shown in Figure 7, the results of the NaIO_3_ group exhibited significantly elevated levels of JNK phosphorylation compared to the normal group (*p* < 0.01 or *p* < 0.05). Interestingly, the pretreatment with ginsenoside Rg3 reduced the expression of p-JNK in the NaIO_3_-stimulated RPE cells (*p* < 0.01 or *p* < 0.05). The findings presented suggest that ginsenoside Rg3 may ameliorate RPE cell damage induced by NaIO_3_ through the inhibition of the JNK signaling pathway. Moreover, the docking simulation results showed that Rg3 interacts with the residues TYR 71, ARG 345, and TYP 71 using conventional hydrogen bonding with the residues TYR 202, ILE 337, LEU 76, and ILE 148, via hydrophobicity interactions forces. Meanwhile, the binding energy of −7.3 kJ/mol also indicates that both of them have good binding ability. Taken together, these results suggest that Rg3 could inhibit RPE cell apoptosis through the inhibition of the JNK pathway.

## 3. Discussion

The sodium iodate (NaIO_3_) model, which exhibits AMD-associated features, has been extensively employed in academic research to enhance the comprehension of the mechanisms underlying cell death in retinal pigment epithelium (RPE) and photoreceptors (PRC). Ginsenoside Rg3, a prominent bioactive compound found in *Panax ginseng*, demonstrates a diverse array of pharmacological properties, encompassing antioxidant, immunomodulatory, anti-inflammatory, and anti-aging activities across various pathological conditions. However, there remains a lack of understanding regarding the therapeutic impact of ginsenoside Rg3 in safeguarding retinal pigment epithelial (RPE) cells from NaIO_3_-induced harm, both in vivo and in vitro. Consequently, this study aims to examine the protective capacity of ginsenoside Rg3 against NaIO_3_-induced retinal injury. Additionally, the potential role of ginsenoside Rg3 in mitigating NaIO_3_-induced ocular damage is also explored. To investigate the impact of ginsenoside Rg3 on the retina, an in vitro and in vivo experimental model of AMD induced by NaIO_3_ was established. In this study, we noted that the intravenous administration of NaIO_3_ at a dosage of 20 mg/kg resulted in retinal pigment epithelium (RPE) degeneration within a period of 7 days. Furthermore, we observed that the total retinal thickness and the thickness of the INL and ONL were significantly reduced at day 7 in the mice treated with NaIO_3_ compared to the control group. By reducing the expression of pro-apoptotic proteins (Bax, cleaved caspase-3, and cleaved caspase-9) and increasing the expression of anti-apoptotic protein (Bcl-2), the P-JNK signaling pathway was also suppressed, alleviating NaIO_3_-induced retinal damage in the mice. It was proven that ginsenoside Rg3 can effectively prevent retinal degeneration caused by sodium iodate.

The existing literature also clearly indicates that large amounts of ROS induce AMD, thereby leading to apoptosis in RPE cells. The retina is particularly vulnerable to oxidative stress due to its continuous exposure to light. This oxidative stress arises when the level of ROS exceeds a specific threshold, triggering a cellular defense mechanism such as autophagy.

In this study, our results indicate that NaIO_3_ can lead to ROS accumulation in RPE cells, resulting in cell death. Our research findings demonstrate that the expression of p-JNK decreases after treatment with ginsenoside Rg3, and the NaIO_3_-induced reduction in Bcl-2 expression and increase in Bax expression are reversed following ginsenoside Rg3 treatment. These results suggest that the anti-apoptotic signals exerted a predominant influence in the group receiving ginsenoside Rg3 treatment. Moreover, the pretreatment with ginsenoside Rg3 resulted in the inhibition of caspase 3 activity, indicating that the administration of ginsenoside Rg3 can potentially improve mitochondrial dysfunction and mitigate the extent of apoptosis. To the best of our understanding, this study provides strong evidence that ginsenoside Rg3 alleviates NaIO_3_-induced retinal degeneration in mice. This study additionally suggests that Rg3 has an anti-Alzheimer’s effect. The completion of this project will lay the foundation for exploring the mechanism of NaIO_3_ multi-organ pathway occurrence.

## 4. Materials and Methods

### 4.1. Reagents and Chemical Compounds 

Ginsenoside Rg3 with a purity > 98% (HPLC method) was prepared as in our previous reported method [19] and purified with high-performance liquid chromatography. Rg3 (3.0 g) with batch number of 2019-01-307-05. It is available in our laboratory. NaIO_3_ (CAS No. 7681-55-2) was obtained from Aladdin with a purity of more than 99% (Shanghai, China). RPE cells were purchased from ATCC Cell Bank. MTT (CAS No. 298-93-1) and Dimethyl sulfoxide (DMSO, CAS No.: 67-68-5) was obtained from Sigma-Aldrich (St. Louis, MO, USA). High-glucose medium, 10% fetal bovine serum (FBS), trypsin, and 100 U/mL penicillin/streptomycin solution were purchased from Logan (UT, USA). Reagent kits, including lactate dehydrogenase (LDH, A020-2-2) and hematoxylin and eosin (H&E, D006-1-1) staining solutions, were supplied by Nanjing Jiancheng Bioengineering Research Institute in Nanjing, China. Dihydroethidium (CAS No. 7681-55-2) was used for the detection of reactive oxygen species, and sourced from Yeasen, Shanghai, China. Immunofluorescence labeling reagents, including SABC-DyLight 488 (SA1094) and Cy3 (SA1074), were procured from BOSTER Bio-Engineer Co., Ltd., in Wuhan, China. Additionally, dye kits for Hoechst 33,258 and JC-1 were acquired for experimental use and the protein quantitation kit (BCA, CAS No.: P0012) was acquired from Beyotime Co., Ltd., in Shanghai, China. Primary antibodies, including Bax, Bcl-2, cleaved caspase 9, caspase 3, cleaved caspase 3, cytochrome C, JNK, and β-actin for Western blotting, were sourced from Cell Signaling Technology in Danvers, MA, USA. Unless otherwise specified, all other reagents and chemicals were procured from Beijing Chemical Factory (Beijing, China).

### 4.2. Animals and Experiment Design 

Eight-week-old male C57BL/6 mice were purchased from LiaoNing ChangSheng (SCXK-2022-0007, Shenyang, China). All mice were raised in a feeding room at a temperature of 23.0 ± 2.0 °C and humidity of 60.0 ± 10.0% with a 12 h light/dark cycle. The mice were housed in a controlled environment with ad libitum access to a standard laboratory diet and water. All procedures involving experimental animals strictly adhered to the Guidelines for the Care and Use of Laboratory Animals at Jilin Agricultural University. The study was approved by the Animal Ethics Committee of Jilin Agricultural University under the Animal Experiment Ethics No. 20220430001. The mice were randomly divided into four groups, with each group containing 12 mice: The Mock group involved animals that received a single intravenous (IV) injection of PBS after an intraperitoneal (IP) injection of PBS. In the Vehicle-treated group, animals were given a single intravenous (IV) injection of 20 mg/kg NaIO_3_ following an intraperitoneal (IP) injection of PBS [20,21]. Animals in the Experimental group were subjected to intraperitoneal (IP) injections of ginsenoside Rg3 at doses of 20 mg/kg and 40 mg/kg, followed by a single intravenous (IV) injection of 20 mg/kg NaIO_3_.

### 4.3. Morphologic Structure of the Retina in AMD Mice

Optical coherence tomography (OCT) was performed using RT Vue XR Avanti with Angio Vue (Optovue Inc., Fremont, CA, USA). After anesthetizing with 15% urethane (urethane, 5 mL/kg), the pupils of the mice were dilated with 0.5% tropicamide (SANTEN, Osaka, Japan). Subsequently, OCT was performed at a certain region of the retina repeatedly, and the resultant scans were examined for changes of the retinal thickness and the retinal microscopic changes.

Fundus images were taken with a Panoramic Ophthalmoscope (Optos 200T×, Dunfermline, UK) without a contact lens and at the indicated wave lengths. After anesthetizing and dilating the pupils, the mice were manually held in front of the Optos, the angle was adjusted, and the fundus images were taken. The retinal vascular changes of the mice were detected by fundus fluorescein angiography (FFA). A dose of 1 mL/100 g body weight of 5% fluorescein sodium was injected into each mouse’s peritoneal cavity. Panoramic Ophthalmoscope was used to continuously focus and capture images of the mouse retina for 5 min, observing the retinal vasculature. Images centered on the optic disc were saved.

### 4.4. Analysis of Histology Changes

Fresh eye tissues were placed in 10% neutral buffered formalin and left at room temperature for over 48 h. Subsequently, the tissues were dehydrated using a series of ethanol solutions, embedded in paraffin, and sliced into 5-micrometer-thick sections using a Leica Rotary Microtome [22] (Leica, RM2245, Shanghai, China). These sections were then subjected to staining with an H&E dye kit, followed by examination and photographing of the resulting pathological changes in the eye tissues using a light microscope [23] (Leica, DM750, Solms, Germany).

### 4.5. RPE Cell Culture

The hTERT RPE-1 cell line (RPE cell line) was provided by the American Type Culture Collection (ATCC). Cultured in DMEM medium supplemented with 10% FBS and 1% penicillin/streptomycin, RPE cells were incubated in a humidified atmosphere with 5% CO_2_ at 37 °C. The medium was replaced every 48 h to maintain cells’ viability until they attained 80–90% confluence.

### 4.6. Cell Viability Assay (MTT Assay)

Cell viability was assessed through MTT assays [24]. Initially, cells were seeded into a 96-well plate at a density of 5 × 10^3^ cells per well, with six replicates per group, and incubated at 37 °C for 24 h. Subsequently, they underwent pretreatment with NaIO_3_ at concentrations of 0.05, 0.1, 0.5, 1, 2.5, 5, 10, 20, and 40 μM for an additional 24 h. Following this, 20 μL of 5 mg/mL MTT solution was introduced into each well, and after a 3.5 h incubation at 37 °C, the culture supernatant was removed. To dissolve formazan crystals, 150 μL of DMSO was added to each well and agitated for 10 min. Ultimately, absorbance at 490 nm was determined using a microplate reader (Nano, Würzburg, Germany), and appropriate concentrations of NaIO_3_ were selected. Similarly, cells were seeded into 96-well culture plates, pretreated with Rg3 at concentrations of 0.5, 1, 2, 4, 8, 16, 32, and 64 μM for 24 h. Subsequent exposure to NaIO_3_ (5 μM) or no exposure occurred for an additional 24 h, and MTT was utilized to evaluate cell survival.

### 4.7. LDH Determination in Cells

Lactate dehydrogenase (LDH) can be released from cells with damaged membranes. Thus, the LDH level in the culture supernatant is a marker indicating the extent of cellular injury [25,26]. RPE cells in the logarithmic growth phase were seeded in 6-well culture plates at a density of 1 × 10^5^ cells per well. To initiate the biochemical testing, the supernatant from RPE cells was combined with the matrix buffer and coenzyme I, following the manufacturer’s instructions. This mixture was then incubated at 37 °C for 15 min. The resultant solutions were introduced to 2,4-dinitrophenylhydrazine, incubated at 37 °C for an additional 15 min, and subsequently treated with 0.4 mol/L NaOH solution. Following thorough mixing, the solutions were allowed to stand at room temperature for 5 min. The absorbance value of the resulting solution was determined at 450 nm using a microplate reader [27] (Nano, Würzburg, Germany). Subsequently, the total cell count in each group was used for normalization.

### 4.8. ROS Staining of Cells

RPE cells in the logarithmic growth phase were seeded in 6-well culture plates at a density of 1 × 10^5^ cells per well. The experiments were conducted when the cell confluence reached 60%. After treatment with varying concentrations (1, 2, and 4 µM) of ginsenoside Rg3 and 5 mM NaIO_3_, the RPE cells were exposed to a 20 μM DHE probe and incubated in the dark at 37 °C for 60 min. Subsequently, the fluorescence intensity was observed using an inverted fluorescence microscope (Leica DM2500, Wetzlar, Germany) to quantify the levels of ROS.

### 4.9. Flow Cytometer Analysis of Apoptosis in RPE Cells

RPE cells in the logarithmic growth phase were seeded in 6-well culture plates at a density of 1 × 10^5^ cells per well. Following the manufacturer’s instructions, RPE cells treated with ginsenoside Rg3 were suspended in the medium and subsequently stained with Annexin V and PI in a light-protected environment. Apoptotic rates were assessed using a flow cytometer with Annexin V-FITC apoptosis assay kits [28] (Sungene Biotechnology, Tianjin, China). The percentages of apoptotic cells were determined using a flow cytometer (FACS Calibur; Becton, Dickinson and Company, Franklin Lakes, NJ, USA).

### 4.10. H&E and Hoechst 33,258 Staining 

RPE cells were initially seeded in a 6-well plate at a density of 1 × 10^5^ cells per well. Once the cell confluence reached 60%, the H&E staining experiment was initiated. In brief, following a 24 h incubation with ginsenoside Rg3 and NaIO_3_, the cells were fixed with 4% paraformaldehyde for 10 min and subsequently permeabilized with 0.2% TritonX-100 for 10 min. The cells were then stained with hematoxylin for 10 min and eosin for 5 min. The resulting stained cells were observed and captured using an inverted optical microscope.

For Hoechst 33,258 staining, a similar approach was adopted to evaluate morphological changes in RPE cells. After a 24 h incubation with ginsenoside Rg3 and NaIO_3_, cells were fixed with 4% paraformaldehyde for 15 min post-fixation, the cells were stained with a 10 μg/mL Hoechst 33,258 staining solution, and the stained nuclei were observed and photographed using an inverted fluorescence microscope [29]. Image-Pro Plus 6.0 image analysis software was employed for quantifying the degree of apoptosis in RPE cells. 

### 4.11. Mitochondrial Membrane Potential (MMP) Assay

To assess the mitochondrial membrane potential of RPE cells, RPE cells in the logarithmic growth phase were seeded in 6-well culture plates at a density of 1 × 10^5^ cells per well. We followed the protocol provided with the JC-1 detection kit [30]. After 24 h of treatment with varying concentrations (1, 2, and 4 µM) of ginsenoside Rg3 and 5 mM NaIO_3_, the cell culture medium was removed. RPE cells were then incubated with 1 mL of JC-1 staining working solution in the dark for 20 min. Following incubation, the supernatant was discarded, and the cells were washed twice with pre-cooled JC-1 staining buffer (1×). Subsequently, the fluorescence staining intensity of the cells was observed and recorded using an inverted fluorescence microscope. The average fluorescence intensity was quantified using Image Pro Plus 6.0 software [31].

### 4.12. Western Blotting Analysis 

RPE cells in the logarithmic growth phase were seeded in 6-well culture plates at a density of 1 × 10^5^ cells per well. To extract total protein from RPE cells, we utilized RIPA lysate along with a protein phosphatase inhibitor. Following extraction, the protein concentration was determined using a BCA protein determination kit, and the samples were prepared for Western blot analysis [32,33]. The protein samples were separated on a 15% SDS-PAGE gel and then transferred to a PVDF membrane. Subsequently, the membrane was blocked using 5% skimmed milk for a duration of 2 h [34,35]. Primary antibodies, including Bax (1:2000), Bcl-2 (1:2000), Cytochrome C (1:1000), caspase 3/9 (1:1000), cl-caspase 3/9 (1:1000), JNK (1:1000), p-JNK (1:1000), and β-actin (1:5000), were incubated with the membrane overnight at 4 °C. After the primary antibody incubation, the membrane was exposed to secondary antibodies for 1.5 h at room temperature [36]. The signals were visualized using an Emitter Coupled Logic (ECL) substrate (Pierce Chemical Co., Rockford, IL, USA). Finally, the intensity of the protein bands was analyzed with Quantity One 4.6.2 software from Bio-Rad Laboratories (Hercules, CA, USA).

### 4.13. Molecular Docking Analysis

First, JNK (PDB ID: 3ELJ) crystal structure was downloaded from PDB. The structure of ginsenoside Rg3 was obtained using the ChemBio Draw 3D tool (Chem3D 21.0.0) [37]. Next, we performed molecular docking using CB-Dock (http://cao.labshare.cn/cb-dock, accessed on 15 May 2024) and visualized the results using BIOVIA Discovery Studio 2019 software [38].

### 4.14. Statistical Analysis 

The data were presented as mean ± standard deviation (mean ± S.D.) and subjected to analysis using one-way analysis of variance (ANOVA). Statistical graphs were generated using GraphPad Prism 8.0.1 software (San Diego, CA, USA). Significance levels were denoted as *p* < 0.001, *p* < 0.01, or *p* < 0.05.

## 5. Conclusions

**Figure 8:**
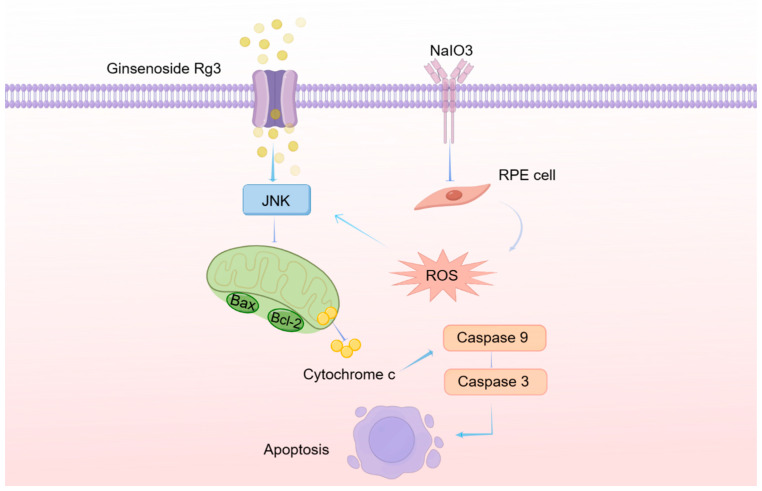
The schematic diagram of molecular mechanism of ginsenoside Rg3 against NaIO_3_-induced ocular toxicity.

In conclusion, our findings indicate that ginsenoside Rg3 mitigates NaIO_3_-induced cell apoptosis by downregulating the expression of Bax, cleaved caspase-3, and cleaved caspase-9, and upregulating the expression of Bcl-2 through the JNK signaling pathway in RPE cells, as shown in Figure 8. Furthermore, in vivo experiments demonstrated that ginsenoside Rg3 has a protective effect against NaIO_3_-induced retinal damage. These results provide support for further exploration of the potential use of ginsenoside Rg3 as a treatment for age-related macular degeneration (AMD).

## Data Availability

The authors confirm that the data supporting the findings of this study are available within the article. The datasets of this study are available from corresponding author upon reasonable request.

## Abbreviations

AMD age-related macular degeneration; NaIO3 sodium iodate; JNK c-Jun N-terminal kinase; P-JNK c-Jun N-terminal kinase phosphorylate; Bax Bcl-2-associated protein; Bcl-2 B-cell lymphoma-2; ROS reactive oxygen species; Caspase-9 cysteinyl aspartate specific proteinase 9; CL-Caspase-9 cleaved cysteinyl aspartate specific proteinase 9; Caspase-3 cysteinyl aspartate specific proteinase 3; CL-Caspase-3 cleaved caspase-3 cysteinyl aspartate specific proteinase 3; INL inner nuclear layer; ONL outer nuclear layer; RPE retinal pigment epithelium.
